# Supplementary material for: Mapping sedentary behaviour (MAPS-B) in winter and spring using wearable sensors, indoor positioning systems, and diaries in older adults who are pre-frail and frail: A feasibility longitudinal study
Source: PLoS One. 2024 May 16;19(5):e0290197. doi: 10.1371/journal.pone.0290197 (PMC11098368; doi:10.1371/journal.pone.0290197)
Supplement: S1 Table — (PDF) [file pone.0290197.s001.pdf]

S1 Table 2007 STROBE Checklist for Cohort Studies

	Item No	Recommendation	Page #
**Title and abstract**	1	(*a*) Indicate the study's design with a commonly used term in the title or the abstract	1, line 1-3
		(*b*) Provide in the abstract an informative and balanced summary of what was done and what was found	2, line 27-47
**Introduction**			
Background/rationale	2	Explain the scientific background and rationale for the investigation being reported	4, line 98-101
Objectives	3	State specific objectives, including any prespecified hypotheses	8, line 208-215
**Methods**			
Study design	4	Present key elements of study design early in the paper	4, line 104-109
Setting	5	Describe the setting, locations, and relevant dates, including periods of recruitment, exposure, follow-up, and data collection	4 & 5, line 111-130
Participants	6	(*a*) Give the eligibility criteria, and the sources and methods of selection of participants. Describe methods of follow-up	5, line 132-140
		(*b*) For matched studies, give matching criteria and number of exposed and unexposed	N/A
Variables	7	Clearly define all outcomes, exposures, predictors, potential confounders, and effect modifiers. Give diagnostic criteria, if applicable	8, line 199-215
Data sources/ measurement	8*	For each variable of interest, give sources of data and details of methods of assessment (measurement). Describe comparability of assessment methods if there is more than one group	6 & 7, line 143-192
Bias	9	Describe any efforts to address potential sources of bias	N/A
Study size	10	Explain how the study size was arrived at	7, line 194-197
Quantitative variables	11	Explain how quantitative variables were handled in the analyses. If applicable, describe which groupings were chosen and why	8, line 218-222
Statistical methods	12	(*a*) Describe all statistical methods, including those used to control for confounding	8, line 218-222
		(*b*) Describe any methods used to examine subgroups and interactions	N/A
		(*c*) Explain how missing data were addressed	8, line 221
		(*d*) If applicable, explain how loss to follow-up was addressed	8, line 221-222
		(*e*) Describe any sensitivity analyses	N/A
**Results**			
Participants	13*	(a) Report numbers of individuals at each stage of study—eg numbers potentially eligible, examined for eligibility, confirmed eligible, included in the study, completing follow-up, and analysed	9, Fig 1
		(b) Give reasons for non-participation at each stage	9, line 229-236
		(c) Consider use of a flow diagram	9, Fig 1
Descriptive data	14*	(a) Give characteristics of study participants (eg demographic, clinical, social) and information on exposures and potential confounders	9, line 239-246, Table 1

		(b) Indicate number of participants with missing data for each variable of interest	10, line 263-265
		(c) Summarise follow-up time (eg, average and total amount)	9, Fig 1
Outcome data	15*	Report numbers of outcome events or summary measures over time	11, line 250-296
Main results	16	(*a*) Give unadjusted estimates and, if applicable, confounder-adjusted estimates and their precision (eg, 95% confidence interval). Make clear which confounders were adjusted for and why they were included	N/A
		(*b*) Report category boundaries when continuous variables were categorized	11, line 250-296
		(*c*) If relevant, consider translating estimates of relative risk into absolute risk for a meaningful time period	N/A
Other analyses	17	Report other analyses done—eg analyses of subgroups and interactions, and sensitivity analyses	N/A
**Discussion**			
Key results	18	Summarise key results with reference to study objectives	13, line 303-316
Limitations	19	Discuss limitations of the study, taking into account sources of potential bias or imprecision. Discuss both direction and magnitude of any potential bias	15, line 386-393
Interpretation	20	Give a cautious overall interpretation of results considering objectives, limitations, multiplicity of analyses, results from similar studies, and other relevant evidence	14, line 352-280
Generalisability	21	Discuss the generalisability (external validity) of the study results	14, line 317-351
**Other information**			
Funding	22	Give the source of funding and the role of the funders for the present study and, if applicable, for the original study on which the present article is based	16, line 408-414

*Give information separately for exposed and unexposed groups.

Note: An Explanation and Elaboration article discusses each checklist item and gives methodological background and published examples of transparent reporting. The STROBE checklist is best used in conjunction with this article (freely available on the Web sites of PLoS Medicine at http://www.plosmedicine.org/, Annals of Internal Medicine at http://www.annals.org/, and Epidemiology at http://www.epidem.com/). Information on the STROBE Initiative is available at http://www.strobe-statement.org.
