# Supplementary material for: Mapping sedentary behaviour (MAPS-B) in winter and spring using wearable sensors, indoor positioning systems, and diaries in older adults who are pre-frail and frail: A feasibility longitudinal study
Source: PLoS One. 2024 May 16;19(5):e0290197. doi: 10.1371/journal.pone.0290197 (PMC11098368; doi:10.1371/journal.pone.0290197)
Supplement: S2 File — (PDF) [file pone.0290197.s003.pdf]

Mapping Sedentary Behaviour (MAPS-B) in Older adults A mix-methods longitudinal study

Principal and lead Investigator

Dr. Isabel B. Rodrigues PhD, MSc

McMaster University, Department of Medicine, Division of Geriatric Medicine, Hamilton, ON

Co-Investigators:

Dr. George Ioannidis[1]
Dr. Jonathan Adachi[1]
Dr. Alexandra Papaioannou[1]
Dr. Dylan Kobsar[2]
Dr. Steven Bray[2]
Dr. Qiyin Fang[3]
Dr. Rong Zheng[4]
Ms. Anne Pizzacalla[5]
Dr. Margaret Denton[5]
Ms. Priscilla Ching[6]

1 McMaster University, Department of Medicine, Hamilton, ON
2 McMaster University, Department of Kinesiology, Hamilton, ON
3 McMaster University, Department of Engineering Physics, Hamilton, ON
4 McMaster University, Department of Computing and Software, Hamilton, ON
5 Hamilton Council on Aging, Hamilton, ON
6 Osteoporosis Canada, Toronto, ON

Funding Sources: New Investigator Fund - Hamilton Health Sciences

BACKGROUND

The global population is living longer and the number of individuals 65 years and older is estimated to reach 2 billion by 2050 (1). A profound implication of the older adult population is the growing number of individuals that present with frailty. Frailty is a multidimensional syndrome characterized by a decline in function across multiple physiological systems including the cardiovascular, musculoskeletal, neurological, and immunological systems (2,3). When exposed to a stressor (e.g., fall, fracture, urinary tract infection), individuals who are frail are more vulnerable to experience a larger and faster deterioration in their health, which may manifest as functional dependency and the inability to return to baseline function and homeostasis (2). Frailty is an important risk factor for negative health outcomes such as increased falls (6,7), fractures (8), hospitalization (8), disability (8), nursing home admission (9), social isolation (4,10), and mortality (11). Frailty is more common in females than in males but mortality rates due to frailty are higher in males, and its prevalence is influenced by socioeconomic factors (12). It is estimated that approximately 1 in 6 community-dwelling older adults may have frailty and it gets worse with age, with 1 in 2 individuals 85 years and older being frail (13).

Older adults who are frail are more likely to be sedentary (14,15). Sedentary behaviour is defined as any waking behaviour in a standing, seating, or reclining posture, with a low energy expenditure (≤ 1.5 metabolic equivalent of task [METS]) (16). The coexistence of frailty and sedentary behaviour worsens overall health (15). Sedentary behaviour is not merely the absence of moderate or vigorous physical activity but also a reduction in sit-to-stand transitions, stand time, and light physical activity (17). Most older adults who are frail spend more than 60% of their awake time in a seated position, which is equivalent to more than 9.5 hours/day of continuous sedentary behaviour (17). The deleterious health effects of sedentary behaviour are different to

those of physical inactivity and are partially independent of an individual's physical activity levels (17). Even older adults who meet the recommended aerobic exercise guidelines of moderate to vigorous physical activity might experience adverse effects of sedentary behaviour (17). Thus, interventions to reduce periods of prolonged sedentary behaviour are necessary.

Older adults who are frail are potentially the population that might benefit the most from a reduction in sedentary time as they are the most sedentary group and have the highest chronic disease burden (18). However, there is a dearth of evidence on effective interventions to reduce sedentary behaviour or total sedentary time in older adults, especially among individuals that are frail (15,19,21). Previous studies focused on reducing total sedentary time, while other studies aimed to increase physical activity levels with the assumption that sedentary time will be reallocated to physical activity (21). But, to date, such interventions have not been effective at reducing total sedentary behaviour time in older adults (15). Previous studies to reduce sedentary time and behaviour in older adults may not be effective because there is no research on the context of sedentary behaviour, about when and where to intervene, and which specific sedentary behaviours should be targeted (15,19). Almost all studies in older adults have assessed total sedentary time, which does not provide enough information to understand the context of sedentary behaviours (15,19). The main reason to understand context is because not all sedentary behaviours should be modified as some cognitively engaging sedentary behaviours (e.g., reading, socializing) appear to benefit health, while time spent in more passive activities may be detrimental. Therefore, the goal should not be to reduce total sedentary time, but rather identify sedentary behaviours that may be detrimental to the health of older adults who are frail (21).

3.0 Objectives and Aims

Before we can develop an intervention to reduce sedentary behaviour among older adults, we must first assess the context of sedentary behaviour. Context is defined as the purpose of the sedentary behaviours, the location where the behaviours occur, the posture of the behaviours (e.g., lying, sitting), social context (e.g., alone or with others), and time of day the behaviours occur. The primary purpose of this study was to assess the feasibility of measuring the context of sedentary behaviour among older adults who are frail. We defined feasibility using recruitment, retention, and refusal rates (process) and the feasibility resource (i.e., can the tools capture context and are participants willing to use the tools). Our criteria for success were to recruit 20 participants within two months, 85% retention, and 20% refusal. Our secondary objectives are: 1) to determine the context of using objective and subjective measures to assess sedentary behaviours among older adults who are frail; 2) to identify which types of sedentary behaviours can be modified and when and where to intervene; and 3) to conduct an exploratory analysis to determine the association of certain types of sedentary behaviours on health-related outcomes. Context is defined as the *purpose* of the sedentary behaviours, the *location* where the behaviours occur, *posture* of the behaviours (e.g., lying, sitting), *social context* (e.g., alone or with others), and *time of day* the behaviours occur.

METHODS

Study Design

We will conduct a one-year mixed-methods longitudinal study with 20 older adults who are frail. To map the context of sedentary behaviour we will use objective (i.e., accelerometers and indoor positioning systems), and self-report (i.e.,diaries) measures; we chose three measures as one measure alone does not provide enough information about context. Participants will be equipped with the accelerometer and indoor positioning system, and complete a diary of daily activities over three days (two weekdays and one weekend) in the winter and spring as sedentary

behaviour may differ by the season. We will also hold focus groups with participants to review the feasibility of using the indoor positioning system and diary and to review the results of the accelerometers, indoor positioning systems, and diaries to identify modifiable sedentary behaviours, and when and where to intervene.

Study Setting

We will collect outcomes at St. Peter's Hospital or in a private clinic in Toronto, Ontario. Data from the accelerometers and indoor positioning systems will be collected remotely from the participant's home and stored on a secure Hamilton Health Sciences and McMaster University server.

Participants & Recruitment

We aim to recruit 20 participants (65 years and older) living with frailty. We will recruit males and females as we anticipate that gender may influence sedentary behaviour through socially constructed norms and roles and can be affected by differential access to resources, opportunities, and power. We are collaborating with geriatricians at Hamilton Health Sciences and in Toronto to recruit participants. The physician will inform eligible participants of our study and provide participants a number to contact the research assistant. The research assistant will speak with interested participants to confirm eligibility and enroll them in the study.

Table 1: Inclusion and exclusion criteria

Inclusion criteria	Exclusion criteria
1. 60 years and older;<br>2. Categorizes as frail on the FRAIL scale ≥ 3 of 5;<br>3. Lives in the Hamilton, Ontario and is a patient at the Hamilton Health Sciences;	1. Requires a wheelchair at least 55% of the awake day; needs to sit for long periods of time due to a medical condition; or not independently mobile (i.e., requires assistance from another individual to ambulate);

4. Speaks English or can attend with a translator.	2. Has travel plans or other commitments that means missing >30% of the rollout study period;<br>3. Unable to follow two-step instructions.

Outcomes

Primary outcome

The primary purpose of this study was to assess the feasibility of measuring the context of sedentary behaviour among older adults who are frail. We defined feasibility using recruitment, retention, and refusal rates (process) and the feasibility resource (i.e., can the tools capture context and are participants willing to use the tools).

<u>Accelerometer:</u> We will use the activPAL4^{TM} accelerometer to collect data on posture and time of day over three consecutive days in the winter and spring. The activPAL4^{TM} is a valid accelerometer in older adults that generates totals for the time spent lying, sitting, standing, and stepping every second of the day (22). The accelerometer is secured to participant's lower thigh with a 3M Tegaderm Transparent bandage and can hold its charge for up to 14 consecutive days. The accelerometer will not interfere with participants daily lives and can be used in the shower or pool. Data is collected wirelessly and stored on the hard drive of the accelerometer and will be transferred to a secured McMaster cloud by a research assistant.

<u>Indoor Positioning System:</u> We will use a McMaster developed indoor positioning system to obtain room/area level positioning information over three days in the winter and spring. The system is designed and validated to be used by older adults in their own homes without the need for a floor plan and only minimal initial setup and calibration (23). The participants will wear a commercially available, off-the-shelf smartwatch with customized software and plug-n-play ambient (nonwearable) beacons in in different rooms of the participant's homes. The smartwatches are

waterproof and can be used in the shower and pool. A typical setting will include one to two watches (depending on participants per household) and three to five Bluetooth beacons per participant. The Bluetooth beacons are labeled with the room's location and plugged into the wall socket (23). The system will detect and track the room-to-room movements of the participants at seconds to minutes intervals (23). Besides location, the system can also measure step counts, heart rate, blood oxygenation, and ambient conditions (lighting, temperature, humidity) (23). The indoor positioning system can be used in homes with multiple stories with multiple residents. The data is collected wirelessly and automatically stored on a secured McMaster cloud. The data can be analyzed through post processing or real-time for acute responses (e.g., falls) (23).

Diary: Each participant will be given an electronic or hard copy of a diary to complete over three days (two weekdays and one weekend during the winter and spring. The diary will ask participants to identify the purpose of the behaviour, the time of day, and social context. To improve diary adherence, we will have a research assistant call the participant the next day to record their 24-hour daily activity using the diary. There is good evidence that reminders improve adherence compared to no reminders (24).

Secondary outcomes

The secondary objectives will be to conduct: 1) focus groups with end-users to identify an intervention on when and where to intervene to breakup or reduce certain types of sedentary behaviour and determine the feasibility of using the indoor positioning system and diary; 2) to determine the context of using objective and subjective measures to assess sedentary behaviours among older adults who are frail; and 3) to conduct an exploratory analysis to determine the association of certain types of sedentary behaviours on health-related outcomes. We will hold two focus groups after the winter and spring collection period to mitigate recall bias. We will use semi-

structured interviews to guide interviews using the Behaviour Change Wheel (25) and the Intersectionality Supplemented Theoretical Domains Framework (26). The purpose of using an intersectional lens is to understand how sedentary behaviour may differ by sex and ethnicity due to gender-constructed norms and cultural differences, respectively. The term intersectionality was coined by professor Kimberlé Crenshaw to describe how gender, race, class, and other individual characteristics "intersect" with one another within structures of power and communities (28). We will also conduct an exploratory analysis to determine the association of certain types of sedentary behaviour on falls, cognition, frailty status, activities of daily living, health-related quality of life, depression, and anxiety (see Table 2).

Table 2: List of secondary outcomes

Secondary outcome	Questionnaire/tool
Fall history	"We would like to know about any falls you have had in the last 6-weeks. Have you had any fall including a slip or trip in which you lost your balance and part or all of your body landed on the floor or ground or lower level?" (29)
Cognitive status	Montreal Cognitive Assessment (MoCA) (30)
Frailty level	Fit-Frailty Assessment and Management Application (App) designed by the GERAS Centre for Aging Research (31)
Activities of daily living	Nottingham Extended Activities of Daily Living Scale (32)
Health-related quality of life	EuroQol 5-Dimension 5-Level (EQ-5D-5L) questionnaire (33)
Depression	Short Form Geriatric Depression Scale (34)
Anxiety	10-Item Geriatric Anxiety Scale (GAS-10) (35)
Demographic characteristics	CIHR endorsed equity lens: Place of residence, Race/ethnicity, Occupation, Gender and sex, Religion, Education, Socioeconomic status, and Social capital (PROGRESS) (36).

Data Collection

We will collect primary and secondary data at two timepoints. Participants will be grouped into four cohorts of 5 participants (total of 20 participants = 5 participants/cohort). During the first week of the winter rollout period, a research assistant will meet with 5 participants at St. Peter's Hospital or in a private clinic in Toronto to equip participants with the accelerometer, indoor positioning

system, and diary, and collect health outcomes (see Table 3). After we collect the primary outcome, the lead investigator will hold a focus group with participants. The same process is repeated for the spring collection period.

Table 3: Data collection for Winter 2023 season

Time	Data collection and timeline
Week 1	Research assistant meets 5 participants (cohort 1) to equip the activPAL4™ and indoor positioning system watch to each participant and ensure it works properly. Participants will also receive their diary and complete several questionnaires (e.g., fall history, cognitive status)
Week 2	Research assistant meets with the 5 participants from week 1 to collect the equipment and diary. The assistant will transfer the data from the activPAL4™ to a computer, and clean and charge the devices.
Weeks 3 to 8	Weeks 1 and 2 are repeated with a new group of 5 participants, until data are collected from 20 participants.
Week 10	Lead investigator holds a focus group with participants.

Sample size

As the primary outcome is qualitative, our sample size is considered large enough to give a spread of experience using this novel approach to mapping sedentary behaviour and context. We anticipate that we will reach saturation at 20 participants.

Ethics

Ethics approval will be obtained from the Hamilton Integrated Research Ethics Board.

Statistical analysis

Primary outcome analysis

Demographic data, feasibility process, and feasibility resources will be reported using means and standard deviations or as a count and percentage. Descriptive analyses were performed using Microsoft Excel (version 16.71). Missing values will be reported as missing. Individuals who were loss to follow-up can be included in the analysis if their data is available. Adverse events will be reported using narrative description.

Secondary outcome analysis

Data analyses from the accelerometer, the indoor positioning system, and participants' diaries will be mapped to the Sedentary behaviour International Taxonomy (SIT) using classification scheme content analysis (37). SIT is a framework developed to help researchers understand the context and determinants of sedentary behaviours. We will post process the data from the accelerometers and indoor positioning system in one-hour intervals. Using posture, we will exclude time intervals related to overnight sleep and walking, and only analyse sedentary behaviours that includes lying, sitting, and standing. Posture, environment, purpose, and social context will be converted to an alphanumeric value based on one of the SIT categories (see Table 4) and we will report the sequences of values as a count/frequency using content analysis. For example, a combination of *lying-bedroom-rest-alone* between 2:30 pm to 3:30 pm would result in a sequence of *P1-E1-U7-S2 in the afternoon*; we will report the most common sequence combinations in the final results.

Table 4: Details on how, when, and where sedentary behaviour will be mapped

<table>
<tr><th>Primary outcome</th><th>SIT Categories to assess primary outcome (37)</th><th>Category Details and sequence codes</th><th>Data Collection Instrument</th></tr>
<tr><td rowspan="6">Context of sedentary behaviour</td><td>Posture</td><td>Lying (P1), sitting (P2), standing (P3), other (P4)</td><td>Accelerometer</td></tr>
<tr><td>Environment</td><td>Bedroom (E1), kitchen (E2), living room/common area (E3), eating area (E4), other (E5)</td><td>Indoor Positioning System</td></tr>
<tr><td>Purpose</td><td>Work (U1), leisure (U2), education (U3), travel (U4), social (U5), eat (U6), rest (U7), care (U8), other (U9)</td><td rowspan="2">Diary</td></tr>
<tr><td>Social Context</td><td>With others (S1), alone (S2)</td></tr>
<tr><td>Measurement</td><td>Objective or self-report</td><td rowspan="2">Accelerometer, Indoor Positioning System, Diary</td></tr>
<tr><td>Time</td><td>Time of day and date</td></tr>
</table>

We will analyze focus group interviews using codebook reliability thematic analysis in Nvivo12 Pro software (QSR International PTY Ltd, Doncaster, Australia). Codes are developed *a priori* from an existing framework, and we will use the Behaviour Change Wheel, which is a commonly used framework to inform interventions and policy changes (25). This framework posits that Capability, Opportunity, and Motivation govern Behaviour (COM-B) (25). Two members of the research team will transcribe and analyze the interviews and classify each theme under a relevant category of COM-B, applying more than one category if needed. The results of focus groups will identify an intervention function and policy change in the Behaviour Change Wheel (See Appendix B).

We will conduct an exploratory analysis of secondary outcomes using multiple linear regression in SPSS Statistics version 27 (IBM Corp, Armonk, New York, USA). Our independent variable will be the most common sequences of sedentary behaviour (e.g., *P1-E1-U7-S2*), and our dependent variable will be the secondary health outcomes (e.g., frailty level, cognitive status). Our covariates will include variables from PROGRESS (i.e., age and sex).

Trial Management and Staff Training

The study central site is at St. Peter's Hospital in Hamilton, ON. The central research assistant and principal investigator will be responsible for ethics, recruitment, and day-to-day trial management. The principal investigator will provide training to the research assistant on how to consent potential participants and collect outcomes (e.g., review the Standard Operating Procedures of each outcome).

Participant Retention and Withdrawal

Once the participant is enrolled, we will make every reasonable effort to follow the participant for the entire study period. The target retention rate will be 85%. Participants may withdraw from

the study for any reason at any time. The principal investigator may also withdraw participants from the study to protect their safety.

Data Management

Data Forms

A research assistant will create all data collection forms using an editable Portable Document Format (PDF). All editable PDFs will be de-identified and we used the participant's assigned ID (e.g., MAPS001, MAPS002).

Data Entry

Data will be entered and managed electronically on an editable PDF, which will be password protected and kept on a secure network system. The MoCA will be completed by the participant using a paper copy and stored at St. Peter's Hospital in a locked cabinet in a locked room that only the lead investigator and research assistant can access. The research assistant will be responsible for entering diary information and secondary outcomes into the editable PDF.

Adverse Events

We will collect adverse events at two times points: during the winter and spring collection periods. We anticipate that the adverse events will not related to the study.

DISCUSSION

Our study is unique because it will be the first study to utilize objective and self-report tools including accelerometers, indoor positioning systems, and diaries to provide context of sedentary behaviours among older adults who are frail. The proposed project is the first step to inform the co-design of an intervention to reduce or breakup sedentary behaviours and may have

enormous economic and social impacts as older adults living with frailty are the highest-cost users of our healthcare system.

References:

1. World Health Organization. Ageing and health [Internet]. Available from: https://www.who.int/news-room/fact-sheets/detail/ageing-and-health
2. Walston J, Hadley E, Ferrucci L, Guralnik J, Newman A, Studenski S, et al. Research agenda for frailty in older adults: toward a better understanding of physiology and etiology: summary from the American Geriatrics Society/National Institute on Aging Research Conference on Frailty in Older Adults. J Am Geriatr Soc. 2006;54(6):991–1001.
3. Abellan van Kan G, Rolland YM, Morley JE, Vellas B. Frailty: toward a clinical definition. Vol. 9, Journal of the American Medical Directors Association. United States; 2008. p. 71–2.
4. Gale CR, Westbury L, Cooper C. Social isolation and loneliness as risk factorsfor the progression of frailty: the English Longitudinal Study of Ageing. Age and Ageing. 2018;47:392–7.
5. Bunt S, Steverink N, Olthof J, van der Schans CP, Hobbelen JSM. Social Frailty Scoping Review .pdf. Eur J Ageing. 2017;14:323–34.
6. Tricco AC, Thomas SM, Radhakrishnan A, Ramkissoon N, Mitchell G, Fortune J, et al. Interventions for social isolation in older adults who have experienced a fall: a systematic review. BMJ Open [Internet]. 2022 Mar 9;12(3):e056540. Available from: https://bmjopen.bmj.com/lookup/doi/10.1136/bmjopen-2021-056540
7. Cheung M, Chang S. Frailty as a Risk Factor for Falls Among Community Dwelling People: Evidence From a Meta-Analysis. J Nurs Scholarsh. 2017;49(5):529–36.
8. Kojima G. Frailty as a predictor of fractures among community-dwelling older people : A systematic review and meta-analysis. Bone. 2016;90:116–22.
9. Godin J, Theou O, Black K, McNeil SA, Andrew MK. Long-term care admissions following hospitalization: The role of social vulnerability. Healthcare (Switzerland). 2019 Sep 1;7(3).
10. Bunt S, Steverink N, Olthof J, van der Schans C, Hobbelen J. Social frailty in older adults: a scoping review. Eur J Ageing. 2017;14(3):323–34.
11. Mitnitski AB, Graham JE, Mogilner AJ, Rockwood K. Frailty, fitness and late-life mortality in relation to chronological and biological age. BMC Geriatr. 2002;2(1):1–8.
12. Zhang Q, Guo H, Gu H, Zhao X. Gender-associated factors for frailty and their impact on hospitalization and mortality among community- dwelling older adults: A cross-sectional population-based study. PeerJ. 2018;2018(2).
13. Ofori-Asenso R, Chin KL, Mazidi M, Zomer E, Ilomaki J, Zullo AR, et al. Global Incidence of Frailty and Prefrailty among Community-Dwelling Older Adults: A Systematic Review and Meta-analysis. JAMA Network Open. 2019 Aug 2;2(8).
14. Petrusevski C, Choo S, Wilson M, MacDermid J, Richardson J. Interventions to address sedentary behaviour for older adults: a scoping review. Vol. 43, Disability and Rehabilitation. Taylor and Francis Ltd.; 2021. p. 3090–101.
15. Chastin S, Gardiner PA, Harvey JA, Leask CF, Jerez-Roig J, Rosenberg D, et al. Interventions for reducing sedentary behaviour in community-dwelling older adults. Cochrane Database of Systematic Reviews. 2021 Jun 25;2021(6).
16. Owen N, Healy GN, Matthews CE, Dunstan DW. Too much sitting: The population health science of sedentary behavior. Exercise and Sport Sciences Reviews. 2010 Jul;38(3):105–13.

17. Blodgett J, Theou O, Kirkland S, Andreou P, Rockwood K. The association between sedentary behaviour, moderate-vigorousphysical activity and frailty in NHANES cohorts. Maturitas. 2015;80(2):187–91.
18. Katzmarzyk PT, Church TS, Craig CL, Bouchard C. Sitting time and mortality from all causes, cardiovascular disease, and cancer. Medicine and Science in Sports and Exercise. 2009 May;41(5):998–1005.
19. Dogra S, Ashe MC, Biddle SJH, Brown WJ, Buman MP, Chastin S, et al. Sedentary time in older men and women: An international consensus statement and research priorities. British Journal of Sports Medicine. 2017 Nov 1;51(21):1526–32.
20. Rawlings GH, Williams RK, Clarke DJ, English C, Fitzsimons C, Holloway I, et al. Exploring adults' experiences of sedentary behaviour and participation in non-workplace interventions designed to reduce sedentary behaviour: A thematic synthesis of qualitative studies. BMC Public Health. 2019 Aug 13;19(1).
21. Copeland JL, Ashe MC, Biddle SJ, Brown WJ, Buman MP, Chastin S, et al. Sedentary time in older adults: A critical review of measurement, associations with health, and interventions. Vol. 51, British Journal of Sports Medicine. BMJ Publishing Group; 2017.
22. Chan CS, Slaughter SE, Allyson Jones C, Ickert C, Wagg AS. Measuring activity performance of older adults using the activpal: A rapid review. Vol. 5, Healthcare (Switzerland). MDPI; 2017.
23. Ianovski A, Fang Q. A Smart Home Platform and Hybrid Indoor Positioning Systems for Enabling Aging in Place. [Hamilton]; 2018.
24. Fenerty SD, West C, Davis SA, Kaplan SG, Feldman SR. The effect of reminder systems on patients' adherence to treatment. Vol. 6, Patient Preference and Adherence. 2012. p. 127–35.
25. Michie S, van Stralen MM, West R. The behaviour change wheel: A new method for characterising and designing behaviour change interventions. Implementation Science. 2011 Apr 23;6(1).
26. Etherington N, Rodrigues IB, Giangregorio L, Giangregorio L, Graham ID, Graham ID, et al. Applying an intersectionality lens to the theoretical domains framework: A tool for thinking about how intersecting social identities and structures of power influence behaviour. BMC Medical Research Methodology. 2020 Jun 26;20(1).
27. Etherington N, Rodrigues I, Ginagregorio L, Graham I, Hoens A, Kasperavicius D, et al. Applying an intersectionality lens to the theoretical domains framework: a tool for thinking about how intersecting social identities and structures of power influence behaviour. BMC Med Res Methodol. 2020;20(169):1–13.
28. Crenshaw K. Demarginalizing the Intersection of Race and Sex: A Black Feminist Critique of Antidiscrimination Doctrine, Feminist Theory and Antiracist Politics. University of Chicago Legal Forum. 1989;1989(1):1–31.
29. Rodrigues IB, Wang E, Keller H, Thabane L, Ashe MC, Brien S, et al. The MoveStrong program for promoting balance and functional strength training and adequate protein intake in pre-frail older adults: A pilot randomized controlled trial. PLoS ONE. 2021 Sep 1;16(9 September).
30. Nasreddine ZS, Phillips NA, Bédirian V, Charbonneau S, Whitehead V, Collin I, et al. The Montreal Cognitive Assessment, MoCA: A Brief Screening Tool For Mild Cognitive Impairment [Internet]. 2005. Available from: www.mocatest.

31. Kennedy C, Opammodos. G, Rockwood K, Thabane L, Adachi J, Kirkland S, et al. A Frailty Index predicts 10-year fracture risk in adults age 25 years and older: results from the Canadian Multicentre Osteoporosis Study (CaMos). Osteoporos Int. 2014;25(12):2825–32.
32. Coventry PA, McMillan D, Clegg A, Brown L, van der Feltz-Cornelis C, Gilbody S, et al. Frailty and depression predict instrumental activities of daily living in older adults: A population-based longitudinal study using the CARE75+ cohort. PLoS ONE. 2020 Dec 1;15(12 December).
33. Xie F, Pullenayegum E, Gaebel K, Bansback N, Bryan S, Ohinmaa A, et al. A Time Trade-off-derived Value Set of the EQ-5D-5L for Canada [Internet]. 2015. Available from: www.lww-medicalcare.com
34. Wancata J, Alexandrowicz R, Marquart B, Weiss M, Friedrich F. The criterion validity of the geriatric depression scale: A systematic review. Vol. 114, Acta Psychiatrica Scandinavica. 2006. p. 398–410.
35. Carlucci L, Balestrieri M, Maso E, Marini A, Conte N, Balsamo M. Psychometric properties and diagnostic accuracy of the short form of the geriatric anxiety scale (GAS-10). BMC Geriatrics. 2021 Dec 1;21(1).
36. O'Neill J, Tabish H, Welch V, Petticrew M, Pottie K, Clarke M, et al. Applying an equity lens to interventions: using PROGRESS ensures consideration of socially stratifying factors to illuminate inequities in health. Journal of Clinical Epidemiology. 2014;67(1):56–64.
37. Chastin S, Schwarz U, Skelton D. Development of a Consensus Taxonomy of Sedentary Behaviors (SIT): Report of Delphi Round 1. PLOS ONE. 2013;8(12):e82313.
38. Hamilton CB, Hoens AM, McQuitty S, McKinnon AM, English K, Backman CL, et al. Development and pre-testing of the Patient Engagement in Research Scale (PEIRS) to assess the quality of engagement from a patient perspective. PLoS ONE. 2018 Nov 1;13(11).
39. Michie S, Atkins L, West R. The behaviour change wheel: a guide to designing interventions. 2nd edn. Silverback Publishing, Sutton; 2014. 59–60 p.

Appendix A: Proposed Study Timeline

	Pre-rollout			Rollout								Data analysis and dissemination		
**Time in months (2022 to 2023)**	**2022**					**2023**								
	**Aug**	**Sept**	**Oct**	**Nov**	**Dec**	**Jan**	**Feb**	**Mar**	**April**	**May**	**Jun**	**Jul**	**Aug**	**Sept**
Principal investigator meets with patient partners from the Hamilton Council on Aging														
Research assistant develops protocol and study materials														
Research assistant completes and submits HiREB application														
Research assistant and collaborators recruits participants														
Research assistant collects Winter outcomes														
Two members of the research team analyses primary outcome (winter)														
Principal investigators holds focus group with research participants and patient partners														
Research assistant collects summer outcomes														
Two members of the research team analyses primary outcome (summer)														
Principal investigators holds second focus group with research participants and patient partners														
Research team analyzes final results and completes manuscript for publication														

Figure A1: Study timeline for the mapping sedentary behaviour project
